# Epidemiology and control of intestinal schistosomiasis on the Sesse Islands, Uganda: integrating malacology and parasitology to tailor local treatment recommendations

**DOI:** 10.1186/1756-3305-3-64

**Published:** 2010-07-27

**Authors:** Claire J Standley, Moses Adriko, Moses Arinaitwe, Aaron Atuhaire, Francis Kazibwe, Alan Fenwick, Narcis B Kabatereine, J Russell Stothard

**Affiliations:** 1 Institute of Genetics, School of Biology, University of Nottingham, NG7 2RD, UK; 2 Wolfson Wellcome Biomedical Laboratories, Department of Zoology, Natural History Museum, Cromwell Road, London, SW7 5BD, UK; 3 Vector Control Division, Ministry of Health, 15 Bombo Road, P.O. Box 1661, Kampala, Uganda; 4 Schistosomiasis Control Initiative, Imperial College London, St Mary's Campus, Norfolk Place, Paddington, London W2 1PG, UK

## Abstract

**Background:**

Intestinal schistosomiasis is often widespread among the populations living around Lake Victoria and on its islands. The Sesse Island group (containing some 84 islands), however, is typically assumed to be a low prevalence zone, with limited transmission, but has never been surveyed in detail. Here, we present a rapid mapping assessment, bringing together snail and parasite information, at 23 sites for the presence of intermediate host snails and at 61 sites for the prevalence of intestinal schistosomiasis in school-aged children (N = 905). Two different diagnostic tools were used and compared at 45 of these sites: Kato-Katz thick faecal smears and circulating cathodic antigen (CCA) urine dipsticks.

**Results:**

*Biomphalaria *snails were found at 11 sites but in low numbers; none was found shedding schistosome cercariae. At 22 out of the 45 sites, local prevalence by urine and/or stool diagnostics was in excess of 50%, although mean prevalence of intestinal schistosomiasis overall was 34.6% (95% confidence intervals (CI) = 31.0-38.3%) by Kato-Katz and 46.5% (95% CI = 42.7-50.4%) by CCA if 'trace' reactions were considered infection-positive (if considered infection-negative, mean prevalence was 28.1% (95% CI = 24.7-31.7%)). Diagnostic congruence between CCA and Kato-Katz was poor and significant discordance in estimated prevalence by location was found, with each often inferring different mass drug administration regimes.

**Conclusions:**

Accurate estimation of schistosome prevalence is important for determining present and future treatment needs with praziquantel; the wide range of schistosome prevalence across the Sesse Island group requires a treatment regime largely tailored to each island. In high prevalence locations, further malacological sampling is required to confirm the extent of local transmission, especially on the northern islands within the group. The observation that different diagnostic tests can provide varying results in terms of estimating prevalence by location, and hence change treatment recommendations, suggests that care must be taken in interpreting raw prevalence data. In particular, further research into the reasons for the differences in the poorer performance of the CCA test should be pursued.

## Background

In Uganda, intestinal schistosomiasis, caused by the trematode parasite *Schistosoma mansoni*, continues to be one of the most important and widespread of the neglected tropical diseases. Its effects are particularly strongly felt in the many communities living around East Africa's Great Lakes, such as the shoreline of Lake Victoria. The distribution of the disease is determined, to a large extent, by the presence or absence of *Biomphalaria *snails, which act as the parasite's intermediate host. However, communities in Lake Victoria have been shown to be highly motile, meaning that a child may be examined for infection at a location other than where the infection was obtained. This emphasises the importance of combining malacological surveys with prevalence spot checks for comprehensive rapid mapping.

Traditional diagnostics used for assessment of schistosome prevalence have relied on stool microscopy, and most commonly, Kato-Katz thick smears. Using stool samples, Lot Quality Assurance Sampling has been tested in Uganda as a cost-effective and rapid sampling method [1]. However, slide preparation is still time-consuming, egg identification requires training and it can be difficult to obtain stool samples on demand, especially if survey time constraints exist. As an alternative, rapid diagnostic tests have been developed to combat these issues for disease surveillance [2,3]. For example, the circulating cathodic antigen (CCA) test (Rapid Medical Diagnostics, Pretoria, South Africa) uses a drop of urine on a lateral flow immuno-chromatographic strip to detect the presence of adult worm infection, and has been successfully tested, in its current formulation, in a highly endemic setting [4]. However, its diagnostic capability has never been examined in a region where the prevalence of intestinal schistosomiasis is suspected to be lower, and infections of more variable intensities.

To combat schistosomiasis in this region, the government of Uganda, with external support, has invested heavily in a national control programme, which has been run by the Vector Control Division (VCD) of the Ministry of Health (with initial support from the Schistosomiasis Control Initiative [SCI] and ongoing funding from RTI International) since 2003 [5,6]. Treatment is largely rolled out through schools, with drug administration intervals determined by prevalence of the disease [7], but there is also presumptive treatment in communities considered at high risk. Until recently, the Sesse Islands were not considered high priority for receiving treatment, however, previous studies have shown that there is significant heterogeneity in schistosomiasis prevalence across the shoreline of Lake Victoria [8]; the Sesse Islands, comprising approximately 9000 km^2 ^of land area over 84 main islands with an estimated population of 44,000 people in 2006, provide an especial challenge in this regard [9]. As such, local-scale mapping is required for accurate determination of a particular locality's drug needs.

Our study therefore attempted to make a rapid mapping assessment of intestinal schistosomiasis on the Sesse Islands and at the same time we compared the diagnostic performance of CCA urine dipsticks with duplicate Kato-Katz thick smears from a single stool (attempts were also made to arbitrate findings against SEA-ELISA serological testing). To assess local transmission, we undertook malacological surveys, where possible, to clarify relationships between disease point prevalence.

## Results

**Figure 1:**
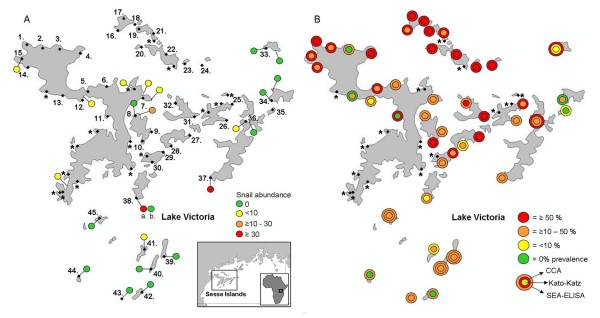
**Results of rapid malacological and parasitological mapping assessment of the Sesse Islands, Uganda, in January 2010**. (a) 23 sites surveyed for *Biomphalaria *snails, with spots coloured by abundance. Site 38 had two habitat types: 'a' denotes a marsh habitat, where *B. sudanica *snails were found, and 'b' denotes a lake habitat, where *B. choanomphala *were found. All other sites were lake habitats. (b) A total of 61 sites where school children were surveyed for intestinal schistosomiasis using Kato-Katz thick smears. Of these, 45 were investigated using both Kato-Katz and CCA urine dipsticks. The remaining sites, where only Kato-Katz thick smears were used, are marked with '*' and the results by location will be presented elsewhere (here the data were only used for overall prevalence estimates). CCA 'trace' results were considered infection-positive here. The location name, geographical coordinates and prevalence of schistosomiasis for each of the 45 sites is reported in Table 1, along with prevalence if CCA 'trace' was considered infection-negative.

The malacological surveys of the islands revealed low abundance of *Biomphalaria *in most of the locations examined (Figure 1a); only one marsh habitat site was found, whereas all the rest were in Lake Victoria proper. On the basis of shell morphology, the *Biomphalaria *from the marsh site (site 38) were putatively identified as *B. sudanica*, whereas all the other *Biomphalaria *observed on this survey were identified as *B. choanomphala*. DNA sequencing of the 'Folmer' fragment of the COI gene of 9 individuals (1 from site 41, 5 from site 38 and 3 from site 37; see Figure 1a) revealed five unique haplotypes (labelled H171-H175; Genbank acquisition numbers HM768902-HM768906), with no cross-over between the populations. Although this is a high level of diversity, divergence was low, with only 0.7% genetic distance across the five haplotypes; this is consistent with on-going molecular research on *Biomphalaria *from elsewhere in Lake Victoria, which suggests that *B. choanomphala *and *B. sudanica *should be considered examples of ecophenotypic variation within a single species or species complex.

None of the snails collected during the surveys were found to be shedding *S. mansoni *cercariae. Rather surprisingly, *B. choanomphala *could be found on rocky and exposed shorelines, on the undersides of pebbles and boulders, and were not restricted to weedy/muddy shorelines as previously thought.

For the parasitological survey, 61 sites were visited in total, and 45 were surveyed using both Kato-Katz and CCA urine lateral flow tests (Figure 1a) with further testing at five of these sites using SEA-ELISA kits. Overall prevalence of intestinal schistosomiasis, as observed via positive Kato-Katz thick smears, was 31.4% (95% confidence intervals [CI] = 28.4-34.5%; number of children surveyed [N] = 905). For the 45 sites surveyed by multiple diagnostics, prevalence by Kato-Katz thick smear was 34.6% (95% CI = 31.0-38.3%; N = 683); when measured by CCA urine lateral flow test, it was 46.5% (95% CI = 42.7-50.4%; N = 666). This was when 'trace' calls were considered infection-positive, as per the manufacturer's instructions. To compare with previous CCA efficacy studies, the prevalence was also calculated for when 'trace' calls were considered negative, and in this case, the value was 28.1% (95% CI = 24.7-31.7%; N = 666). Prevalence ranged widely across the islands, with high prevalence (> 50%) localities predominant in the northern islands of Bufumira sub-county (Figure 1b). A full list of the schools visited, with their geographical coordinates, number of children surveyed and prevalence by both Kato-Katz thick smear and CCA test, can be found in Table 1.

**Table 1:** Map ID number, GPS coordinates, village name, subcounty and prevalence of intestinal schistosomiasis as determined by Kato-Katz and CCA tests. 'N' = number of children surveyed; 'CI' = confidence intervals.

Map ID (***GPS coordinates)***	Village	Subcounty	Prevalence by Kato-Katz	Prevalence by CCA	
			(N, 95% CI)	**'Trace' = pos**. (N, 95% CI)	**'Trace' = neg**. (N, 95% CI)
1 *(S0.22048, E32.07748)*	Kibanga	Mugoye	68.8 (16, 41.3-89.0)	100.0 (16, 79.4-100.0)	68.8 (16, 41.3-89.0)
2 *(S0.22820, E32.10802)*	Buziga	Mugoye	46.2 (13, 19.2-74.9)	56.3 (16, 29.9-80.2)	25.0 (16, 7.3-52.4)
3 *(S0.22986, E32.14310)*	Bungo	Mugoye	0.0 (12, 0.0-26.5)	15.4 (13, 1.9-45.4)	0.0 (13, 0.0-24.7)
4 *(S0.23807, E32.18468)*	Kasekulo	Mugoye	50.0 (16, 24.7-75.3)	62.5 (16, 35.4-84.8)	43.8 (16, 19.8-70.1)
5 *(S0.31496, E32.19963)*	Kagolomola	Mugoye	14.3 (14, 1.8-42.8)	100.0 (14, 76.8-100.0)	50.0 (14, 23.0-77.0)
6 *(S0.30533, E32.23756)*	Kasenyi	Mugoye	16.7 (12, 2.1-48.4)	13.3 (15, 1.7-40.5)	6.7 (15, 0.2-31.9)
7 *(S.32837, E32.31014)*	Mwena	Mugoye	21.4 (14, 4.7-50.8)	28.6 (14, 8.4-58.1)	7.1 (14, 0.2-33.9)
8 *(S0.36381, E32.29543)*	Kaagonya	Mugoye	25.0 (8, 3.2-65.1)	12.5 (8, 0.3-52.7)	12.5 (8, 0.3-52.7)
9 *(S0.39600, E32.32784)*	Kisujju	Mugoye	28.6 (14, 8.4-58.1)	46.2 (13, 19.2-74.9)	46.2 (13, 19.2-74.9)
10 *(S0.41439, E32.30922)*	Kivunza	Mugoye	77.8 (9, 40.0-97.2)	66.7 (9, 30.0-92.5)	66.7 (9, 30.0-92.5)
11 *(S0.36485, E32.23876)*	Njoga	Mugoye	0.0 (14, 0.0-23.2)	100.0 (15, 78.2-100.0)	0.0 (15, 0.0-21.8)
12 *(S0.33256, E32.18855)*	Banga	Mugoye	6.7 (15, 0.2-31.9)	11.8 (17, 1.5-36.4)	11.8 (17, 1.5-36.4)
13 *(S0.32341, E32.14947)*	Mutambala	Mugoye	10.0 (10, 0.3-44.5)	0.0 (14, 0.0-23.2)	0.0 (14, 0.0-23.2)
14 *(S0.26678, E32.07942)*	Banda1	Mugoye	28.6 (14, 8.4-58.1)	64.3 (14, 35.1-87.2)	42.9 (14, 17.7-71.1)
15 *(S0.24923, E32.06704)*	Luku	Mugoye	47.1 (17, 23.0-72.2)	58.8 (17, 32.9-81.6)	35.3 (17, 14.2-61.7)
16 *(S0.19225, E32.25963)*	Luwungulu	Bufumira	30.8 (13, 9.1-61.4)	50.0 (14, 23.0-77.0)	21.4 (14, 4.7-50.8)
17 *(S0.16808, E32.27124)*	Kammesse	Bufumira	73.3 (15, 44.9-92.2)	93.3 (15, 68.1-99.8)	66.7 (15, 38.4-88.2)
18 *(S0.17993, E32.29384)*	Kachanga	Bufumira	75.0 (16, 47.6-92.7)	100.0 (15, 78.2-100.0)	73.3 (15, 44.9-92.2)
19 *(S0.18954, E32.30225)*	Kaaya	Bufumira	66.7 (18, 41.0-86.7)	100.0 (15, 78.2-100.0)	60.0 (15, 32.3-83.7)
20 *(S0.22574, E32.30759)*	Kibibi	Bufumira	57.1 (7, 18.4-90.1)	57.1 (7, 18.4-90.1)	42.9 (7, 9.9-81.6)
21 *(S0.19813, E32.33113)*	Misonzi	Bufumira	46.9 (32, 29.1-65.3)	73.3 (15, 44.9-92.2)	53.3 (15, 26.6-78.7)
22 *(S0.24036, E32.35637)*	Bosa	Bufumira	52.9 (17, 27.8-77.0)	71.4 (14, 41.9-91.6)	35.7 (14, 12.8-64.9)
23 *(S0.25941, E32.39413)*	Banda	Bufumira	73.3 (15, 44.9-92.2)	93.3 (15, 68.1-99.8)	86.7 (15, 59.5-98.3)
24 *(S0.26246, E32.42848)*	Kitobo	Bufumira	69.2 (26, 48.2-85.7)	80.0 (15, 51.9-95.7)	60.0 (15, 32.3-83.7)
25 *(S0.34038, E32.48982)*	Namasengere	Bufumira	37.5 (16, 15.2-64.6)	92.9 (14, 66.1-99.8)	50.0 (14, 23.0-77.0)
26 *(S0.37258, 32.47824)*	Mukaka	Bufumira	46.7 (15, 21.3-73.4)	33.3 (15, 11.8-61.6)	6.7 (15, 0.2-31.9)
27 *(S0.40281, E32.40434)*	Kaazi	Bufumira	86.7 (15, 59.5-98.3)	80.0 (15, 51.9-95.7)	53.3 (15, 26.6-78.7)
28 *(S0.42411, E32.36667)*	Kyankolokolo	Bufumira	20.0 (15, 4.3-48.1)	6.7 (15, 0.2-31.9)	6.7 (15, 0.2-31.9)
29 *(S0.43715, E32.35111)*	Lwabaswa	Mugoye	18.2 (11, 2.3-51.8)	84.6 (13, 54.6-98.1)	76.9 (13, 46.2-95.0)
30 *(S0.45702, E32.32824)*	Kasisa	Mugoye	25.0 (12, 5.5-57.2)	45.5 (11, 16.7-76.6)	36.4 (11, 10.9-69.2)
31 *(S0.36561, E32.41432)*	Lulindi	Bufumira	55.6 (18, 30.8-78.5)	66.7 (12, 34.9-90.1)	50.0 (12, 21.1-78.9)
32 *(S0.33807, E32.37603)*	Ssemawundo	Bufumira	59.4 (32, 40.6-76.3)	46.7 (15, 21.3-73.4)	40.0 (15, 16.3-67.7)
33 *(S0.22757, 32.55733)*	Jaana	Jaana	5.0 (20, 0.1-24.9)	8.7 (23, 1.1-28.0)	4.3 (23, 1.1-21.9)
34 *(S0.33335, E32.56855)*	Bubeke	Jaana	23.1 (13, 5.0-53.8)	0.0 (16, 0.0-20.6)	0.0 (16, 0.0-20.6)
35 *(S0.35153, E32.57128)*	Buyange	Kyamuswa	0.0 (17, 0.0-19.5)	6.3 (16, 0.2-30.2)	0.0 (16, 0.0-20.6)
36 *(S0.37463, E32.51683)*	Lwenabatwa	Kyamuswa	10.0 (10, 0.3-44.5)	40.1 ( 23, 19.7-61.5)	13.0 (23, 2.8-33.6)
37 *(S0.48870, E32.44622)*	Nakibanga	Kyamuswa	26.7 (15, 7.8-55.1)	13.3 (15, 1.7-40.5)	13.3 (15, 1.7-40.5)
38 *(S0.52895, E32.29731)*	Mawala	Mazinga	7.7 (13, 0.2-36.0)	20.0 (15, 4.3-48.1)	20.0 (15, 4.3-48.1)
39 *(S0.64666, E32.35232)*	Kuuye	Mazinga	20.0 (20, 5.7-43.7)	25.0 (20, 8.7-49.1)	15.0 (20, 3.2-37.9)
40 *(S0.67167, E32.32633)*	Kachungwa	Mazinga	10.0 (20, 1.2-31.7)	13.0 (23, 2.8-33.6)	4.3 (23, 0.1-21.9)
41 *(S0.63200, E32.31278)*	Kirugu	Mazinga	14.3 (14, 1.8-42.8)	5.9 (17, 0.1-28.7)	5.9 (17, 0.1-28.7)
42 *(S0.71890, E32.30853)*	Nkose	Mazinga	0.0 (13, 0.0-24.7)	15.4 (13, 1.9-45.4)	7.7 (13, 0.2-36.0)
43 *(S0.73168, E32.26802)*	Miyana	Mazinga	12.5 (8, 0.3-52.7)	28.6 (7, 3.7-71.0)	0.0 (7, 0.0-41.0)
44 *(S0.68592, E32.18170)*	Lujjabwa	Mazinga	0.0 (14, 0.0-23.2)	35.3 (17, 14.2-61.7)	0.0 (17, 0.0-19.5)
45 *(S0.56909, E32.22253)*	Butulume	Mazinga	13.3 (15, 1.7-40.5)	26.7 (15, 7.8-55.1)	0.0 (15, 0.0-21.8)

**TOTAL**	**--**	**--**	**34.6** **(683, 31.0-38.3)**	**46.5** **(666, 42.7-50.4)**	**28.1** **(666, 24.7-31.7)**

For the five villages surveyed with Kato-Katz, CCA and SEA-ELISA, overall prevalence by each of the three diagnostics was 11.7% (95% CI = 5.8-20.6%; N = 85), 22.1 (95% CI = 14.6-31.3%; N = 104) and 39.2% (95% CI = 29.7-49.4%; N = 102) respectively [Table 2]. In all sites bar one, SEA-ELISA resulted in the highest prevalence. Prevalence when CCA traces were considered infection-positive varied widely compared to when they were calculated as being infection-negative.

**Table 2:** Prevalence of schistosomiasis comparing Kato-Katz diagnostic with CCA tests and SEA-ELISA

Site (Map ID)	DIAGNOSTIC TEST									
	
	Kato-Katz			CCA				SEA-ELISA		
	
	N	Infection intensity L/M/H	Prevalence (95% CIs)	N	Infection intensity tr/+/++/+++	Prevalence - trace as positive (95% CIs)	Prevalence - trace as negative (95% CIs)	N	Infection intensity tr/+/++/+++	Prevalence (95% CIs)
Butulume (45)	15	2/0/0	13.3 (1.7-40.5)	15	4/0/0/0	26.7 (7.8-55.1)	0.0 (0.0-21.8)	15	3/0/0/0	20.0 (4.3-48.1)

Jaana (33)	20	1/0/0	5.0 (0.1-24.9)	23	1/1/0/0	8.7 (1.1-28.0)	4.3 (0.1-21.9)	22	0/6/2/4	54.5 (32.2-75.6)

Kachungwa (40)	20	2/0/0	10.0 (1.2-31.7)	23	2/1/0/0	13.0 (2.8-33.6)	4.3 (0.1-21.9)	23	2/1/4/2	39.1 (19.7-61.4)

Kuuye (39)	20	3/1/0	20.0 (5.7-43.7)	20	2/2/1/0	25.0 (8.7-49.1)	15.0 (3.2-37.9)	20	1/1/3/1	30.0 (11.9-54.3)

Lwenabatwa (36)	10	1/0/0	10.0 (0.3-44.5)	23	6/2/1/0	39.1 (19.7-61.5)	13.0 (2.8-33.6)	22	2/2/3/6	59.1 (36.4-79.3)

**TOTAL**	85	9/1/0	11.7 (5.8-20.6)	104	15/6/2/0	22.1 (14.6-31.3)	7.8 (3.3-14.6)	102	8/10/12/13	39.2 (29.7-49.4)

'Trace' read for CCA tests considered both as positive and negative. 'N' refers to the number of children surveyed by each diagnostic; 'L' stands for 'Light' infection intensity (< 100 eggs per gram of faeces [epg]), 'M' corresponds to 'Moderate' infection intensity (≥100 < 400 epg) and 'H' refers to 'Heavy' infection intensity (≥ 400 epg). 'tr' stands for trace reaction of CCA test, and '+', '++' and '+++' stand for single, double and triple strength reactions, respectively.

**Figure 2:**
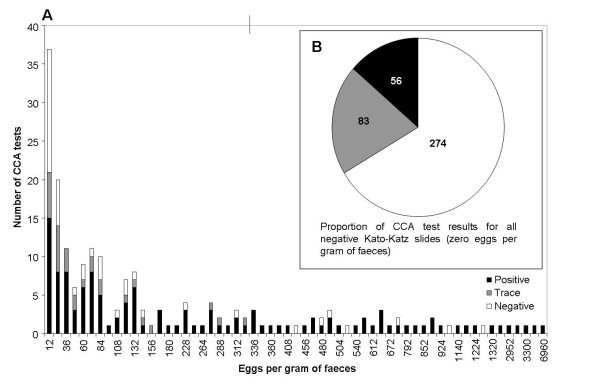
**Comparison of egg per gram (EPG) scores and corresponding CCA test result as 'positive', 'trace' or negative**. (a) Number of CCA tests for a particular EPG score, and the breakdown of the tests into 'positive', 'trace' and 'negative' results. (b) Breakdown of CCA test results for schoolchildren who had zero *S. mansoni *eggs detected in their Kato-Katz thick smears.

**Figure 3:**
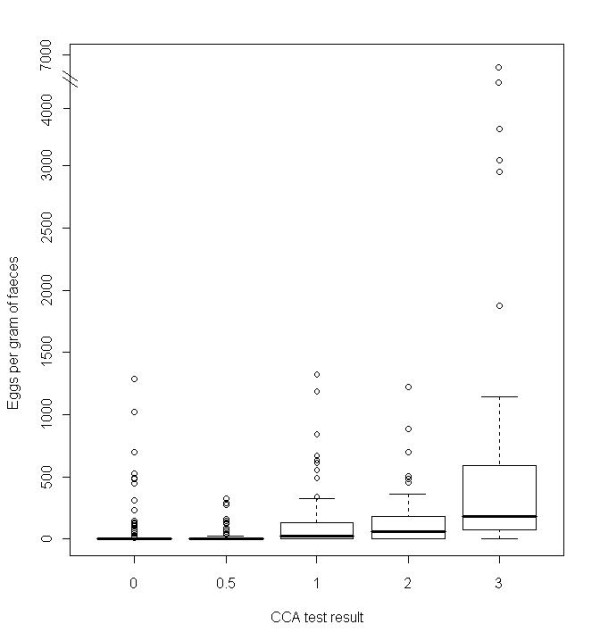
**Box plot of CCA test band strength against *S. mansoni *egg per gram count**. Egg count based on duplicate Kato-Katz thick smear; indents in the boxes denote 95% CI.

The relationship between Kato-Katz thick smear result and CCA was investigated further by directly comparing the CCA test result of children with a particular faecal egg per gram (EPG) value (Figure 2). A large number (139 out of 413) of children with no eggs observed in microscopic examination of their stool had positive or 'trace' CCA tests; similarly, an individual with an egg count as high as 1284 EPG had a negative CCA result. These outliers can also be seen in the box plot measuring CCA test score against EPG directly (Figure 3). Accordingly, diagnostic scores were low when comparing the CCA tests, calculated both for 'trace' being positive as well as 'trace' being negative, against Kato-Katz values (Table 3).

**Table 3:** Diagnostic scores of CCA test against 'gold standard' of double Kato-Katz smear

Trace call	SS (95% CIs)	SP (95% CIs)	PPV (95% CIs)	NPV (95% CIs)
**Positive**	78.2 (72.1-83.5)	66.3 (61.7-70.8)	52.6 (46.9-58.2)	86.4 (82.4-89.9)

**Negative**	63.5 (56.6-69.9)	86.4 (82.9-89.5)	69.1 (62.1-75.5)	83.2 (79.5-86.5)

'Positive' refers to 'trace' calls on the CCA test being considered positive, whereas 'Negative' refers to counting 'trace' results as negative.

**Figure 4:**
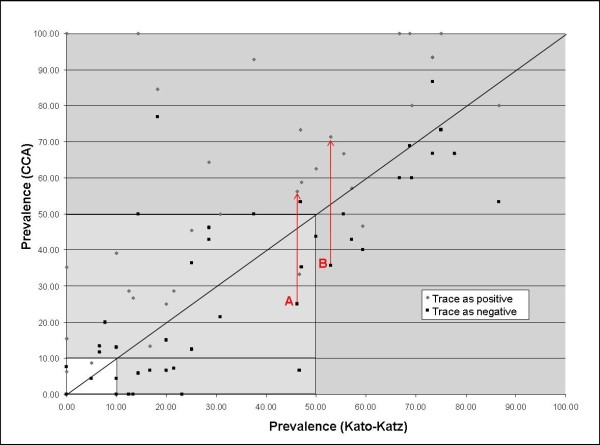
**Scatter plot of prevalence as determined by Kato-Katz against that determined by CCA test**. 'Trace' result as negative is compared to 'trace' result as positive. The graph area is shaded based on the WHO thresholds for different treatment recommendations. 'A' denotes a site where Kato-Katz and CCA (when 'trace' results are called negative) both show prevalence under 50%, but which rises to over 50% if 'trace' CCA calls are considered positive. 'B' denotes a site where Kato-Katz and CCA results agree on a prevalence but only when CCA 'trace' results are considered positive; if considered negative, CCA result drops below 50%.

Prevalence at each school as determined by Kato-Katz and by CCA urine test was directly compared with a scatter plot, which further compared the difference in prevalence value as obtained when 'trace' calls were infection-positive and when they were infection-negative (Figure 4). As treatment regimes are based on prevalence categories, these were also included on the figure. The lines marked 'A' and 'B' refer to cases whereby downgrading 'trace' calls from positive to negative drops that particular school under 50% prevalence, thus changing its recommended treatment regime. For 'A', the Kato-Katz prevalence is below 50%, thus agreeing with the 'trace' as negative CCA test result; however, for 'B', Kato-Katz prevalence was above 50%. Table 4 shows a breakdown of the recommended treatment regimes for each location, based on these surveys, and further demonstrates the number of sites that would change treatment regimes either between the Kato-Katz and CCA results, or even between calling 'trace' results as positive and calling them as negative. It is of note that in some low-to-moderate prevalence settings, CCA dipsticks have underestimated treatment needs by location, when compared to Kato-Katz examinations, resulting in conflicting mass drug administration regimes.

**Table 4:** Number of sites in each treatment strategy classification, based on different diagnostics

	No treatment (prevalence < 10%)			Single treatment (≥ 10-50%)			Annual treatment (≥ 50%)		
	**Kato-Katz**	**CCA (trace = positive)**	**CCA (trace = negative)**	**Kato-Katz**	**CCA (trace = positive)**	**CCA (trace = negative)**	**Kato-Katz**	**CCA (trace = positive)**	**CCA (trace = negative)**

**Number of sites**	9	6	16	23	18	16	13	21	13

## Discussion

### Mapping schistosomiasis in the Sesse Islands

Mapping this island group for schistosomiasis poses a significant logistical challenge in terms of equipment and resources as only the main island (Kalangala) is directly accessible by ferry from the mainland, whilst all the others are reachable by small boat access alone. Many of the Sesse Islands, especially those in the south-east, are remote and poorly served, yet are inhabited by large numbers of people (and associated communities) typically engaged in the Lake Victoria fishery. Our survey has shown that far from being a uniformly low transmission zone, as had been assumed previously, the Sesse Islands display significant heterogeneities in terms of the prevalence of *S. mansoni*, with obvious implications for on-going treatment campaigns and revision of general advice for tourists visiting these islands. Rather surprisingly, the CCA urine lateral flow tests did not perform as well in this putative low transmission setting, in terms of diagnostic scores as compared to Kato-Katz thick smears, as had been the case in other regions of Lake Victoria where transmission was high [4].

The prevalence maps of the Sesse Islands clearly show that the northern islands in the archipelago, in northern Bufumira sub-county, appear a local hot spot for schistosomiasis. Much of the rest of the archipelago, and especially the southern and eastern out-lying islands, have much lower prevalence of the disease; these areas were also surveyed for snails, and *Biomphalaria *abundance observed to be low, although not absent. Unfortunately, owing to time and personnel constraints, snail surveys were not undertaken in Bufumira sub-county; therefore we cannot yet know whether the high prevalence observed there could be maintained through local transmission, or whether the children had acquired their infections elsewhere. Previous demographic surveys in Lake Victoria have revealed very high levels of itinerancy among the communities living along the lakeshore; in Kalangala District, in 2008, almost 60% of the 189 children surveyed had been born outside of the district, and as far afield as Tanzania [8]. The World Health Organization has put a large emphasis on the need to create predictive maps, for example of expected schistosome distributions, but these need to be validated on the ground; Bufumira sub-county would be a crucial location for further malacological surveys in order to better understand the relationship between prevalence and snail distribution and thus create more accurate predictive maps. Vector Control Division will shortly be providing an updated atlas of the prevalence of intestinal schistosomiasis across the shoreline of Lake Victoria drawing further attention to disease heterogeneity at the local level.

### Diagnostic comparisons and differences

Across the district, a number of sites had significant differences in the prevalence as ascertained by one diagnostic or another. For example, Site 11 would have been considered high prevalence on the basis of CCA results, and yet not a single positive Kato-Katz thick smear was observed. Conversely, two sites would have been considered absent of *S. mansoni *had only CCA tests been used, and yet had Kato-Katz prevalence of 23.1% and 10.0% respectively.

The variability of the CCA test in this setting is the cause of some concern, and indeed the diagnostic scores are generally lower than given in other studies [10-12]. Moreover, the results could be explained by the particular transmission dynamics of schistosomiasis in these islands. As we have shown, in many localities in the Sesse Islands there is a relative lack of intermediate host snails, as compared to other parts of the Ugandan shoreline. If the Sesse Islands themselves are not a high transmission zone, children may only be exposed to the parasite infrequently, resulting in long-term infection from single or infrequent exposure event(s). As these worms age, fecundity could decrease with senescence, reducing egg output intensity and in this scenario, a CCA test would be positive whereas the Kato-Katz thick smear might not reveal eggs.

Another factor to consider is that the CCA test is thought to be sensitive to differences in antigenicity of the parasite populations [3,13]; it is plausible that changes in parasite genotypes (and phenotypes) across the lakeshore may result in altered performance of the CCA test. Similarly, if exposure is low, the diversity of the parasite genotypes present within an individual child may also be lower than in other regions, which too could alter the way in which the test reacts.

The SEA-ELISA results potentially reveal a further complication; the effect of prior treatment. As the egg antigens used in the ELISA may persist after the parasite is eliminated, for example post-treatment with praziquantel, individuals may test sera-positive despite not having an active infection. In the Sesse Islands, while treatment is far from ubiquitous, the district has been involved in the national control programme since 2005, and mass drug administration has been underway since then, particularly on the main island. In this survey, 49 out of 226 children (21.7%) interviewed on site reported having received praziquantel at some point. As such, whereas SEA-ELISA testing is an extremely sensitive and effective way of testing baseline prevalence in an un-surveyed area, its lack of discrimination between historical and current infections make it unsuitable for on-going monitoring, particularly where a treatment programme is in place.

### Implications for treatment recommendations

The main aim in measuring prevalence of schistosomiasis in these various locations is to use the information to provide recommendations to the Vector Control Division's Ministry of Health, to streamline the delivery of praziquantel in their national treatment programme and to maximise its therapeutic coverage across the disease endemic landscape. Under-estimating the prevalence of intestinal schistosomiasis by locality leads to incorrect assessment of actual treatment needs, and since drug resources are finite, medications could be reassigned to other sites incorrectly. Conversely overestimating the prevalence of the disease by site could lead to increased drug wastage as treatments may be given to patients when not actually needed, but ethically, overtreatment is a more equitable position than omission of treatment [14].

Here, we have shown that SEA-ELISA is likely to overestimate present treatment needs as past treatment history likely confounds this assay, particularly in areas where mass drug distribution has taken place. However, we also show that CCA tests can often inaccurately estimate prevalence, at least when compared against Kato-Katz thick smears from a single stool sample. Nonetheless, the need for mapping prevalence can bring forward significant drug savings, as well as programmatic costs when set across a 5-year period of intervention. The Sesse Islands can be considered a further example of where good baseline information is needed to guide sensible decisions in meeting present and future local treatment needs. In this setting, and as a rough approximation, the raw costs of blanket annual mass drug administration for 5 years could be estimated as US$7593 (45 villages each having 250 children per village with an average dose of 2.7 tablets of praziquantel per child at US$0.05 per tablet). Simply by doing a local-scale rapid assessment survey such as this one, costs of drug procurement can be more than halved. Based on Kato-Katz prevalence, 13 schools require annual treatment; if the other 32 are all given a single dose, the cost is now US$3273.75, for the 5-year period. This value holds true for if 'trace' CCA calls are considered negative, as well. If 'trace' is considered infection-positive, the amount needed rises to US$4353.75, but this is still much less than the cost of blanket annual treatment. Of course, this does not take into account the cost of training, transport, monitoring and other supplementary activities. Nevertheless the point stands: regardless of diagnostic technique, implementing local-scale monitoring into control programme surveys can greatly reduce the cost of the intervention, by tailoring treatment regimes to local needs especially from longer term perspectives and provide evidence-based decision making.

As we have discussed, however, the difficulty lies with determining exactly what local treatment needs actually are, especially in the face of varying results from different diagnostics. Here, more thematic research is needed, for example in comparing the CCA tests against triplicate Kato-Katz thick smears from successive stools, as a more accurate 'gold' measure. Doing this could lead to a correction factor for the CCA test, to be used in rapid assessment settings where only single stool samples are examined. Alternatively, other eggs in stool detection methods could be explored, some of which have been shown to be potentially more sensitive than triple Kato-Katz thick smears for detecting parasite eggs [15,16]. However, all alternative methods should be field-tested to determine efficacy and usability, even in remote settings, before being widely recommended and scaled-up for rapid mapping endeavours [17]. For the meantime, we urge researchers to continue to test the CCA urine lateral flow test alongside Kato-Katz thick smears, in different epidemiological settings which may later allow a future meta-analysis of performance across transmission landscapes. In addition, it would be useful to explore in greater detail the effect of parasite genetic diversity on the diagnostic sensitivity of CCA tests; as our knowledge base grows regarding the molecular epidemiology of *S. mansoni *in Lake Victoria, we may be better placed to judge its effect on such diagnostic tools.

## Conclusions

Here, we present the first comprehensive survey of intestinal schistosomiasis and intermediate host snails on the Sesse Islands, in Lake Victoria. Long considered a low priority for treatment interventions, we have shown that in fact prevalence in some areas is very high, and certainly sufficient to merit on-going annual treatment. We also demonstrate the need for more rigorous malacological sampling across the archipelago, and particularly in high prevalence environments. However, we also show that treatment recommendations can vary depending on the diagnostic test used; these differences will need to be examined more closely to ensure that the efficacy and efficiency of the control programme is maximised.

## Methods

### Malacological surveys

Snail surveys were undertaken in 23 locations around the Sesse Islands in 2008 and 2010. Sampling was semi-quantitative, with two collectors scooping for a period of 20 min over an area of shoreline approximately 50 m in length. Habitat type was noted, and a putative field identification made of the *Biomphalaria *snails collected, based on shell morphology. Water chemistry values, such as pH, conductivity (μS) and total dissolved solutes (TDS) were also taken. Geographical position (GPS coordinates) of the site was recorded using a Garmin GPS V^® ^(Garmin (Europe) Ltd.; Southampton, UK).

DNA from 24 snails from four locations was extracted using a standard CTAB and chloroform-isoamyl alcohol methodology, with precipitation in 100% ethanol and final rehydration in pure water. PCR amplifications were carried out on a Veriti thermal cycler (Applied Biosystems Inc., Foster City, CA, USA) using the 'Folmer' universal primers and according to published cycling conditions [18]; all products were visualised on 1% agarose gel stained with GelRed™(Hayward, CA, USA). The amplification reactions were done in 25 μl total volume, with 2.5 μl MgCl_2 _(20 MM concentration), 2.5 μl 5 × buffer, 2.5 μl pre-mixed dNTPs (20 MM concentration), 1 μl each of forward and reverse primer (10 pmol concentration) and one unit of TAQ per reaction. The 22 successfully amplified PCR products were purified using a QIAQuick PCR Purification Kit (QIAGEN Ltd, Crawley, UK) and product concentration was quantified on a Nanodrop ND-1000 Spectrophotometer (Nanodrop Technologies Inc., Willington, USA). Sequencing reactions were performed on mitochondrial purified PCR products using an Applied Biosystems Big Dye Kit (version 1.1) and run on an Applied Biosystems 3730 DNA Analyzer (Applied Biosystems, Carlsbad, USA). Sequences were assembled and edited by eye using Sequencher v 4.8 (Gene Codes Corporation, Ann Arbor, Michigan, USA: http://www.genecodes.com); 9 double-stranded sequences were able to be edited clearly and these were used in later analysis. Distance calculations were performed in MEGA 4.0 [19].

### Parasitological surveys

In total, 1014 children were sampled from 61 localities across the Sesse Islands (Kalangala district). 514 were male and 500 were female, and the mean age was 9.5 years (see below for details of data cleaning prior to analysis). 45 sites (numbered on Figure 1a) were sampled using both Kato-Katz thick double smears, from a single stool, and CCA urine lateral flow tests (Rapid Medical Diagnostics, Pretoria, South Africa). As an alternative arbitration of diagnostic results, at 5 of these sites (Figure 1b) children were also tested on site for antibodies against soluble egg antigens using SEA-ELISA kits (IVD Inc.; Carlsbad, USA) with sera collected from fingerprick blood and processed according to kit instructions. GPS coordinates were also recorded for each parasitological survey location (as above).

### Sample collection and preparation

Stool and urine samples were collected with the assistance of local community health officers, mobilisers and Vector Control Division technicians. Finger-prick blood was collected by a trained community nurse, and consumables disposed of immediately following the SEA-ELISA tests. Approximately 0.5 mol of finger-prick blood was centrifuged to separate out the serum; this was used in the SEA-ELISA test at a dilution of 1:40 (as per the manufacturer's instructions).

Stool samples were used to prepare a duplicate Kato-Katz thick smear on a single microscope slide, as per standard methods [20]. These slides were read *in situ *after a clearing period of approximately 30 min and counted for *S. mansoni *eggs. Slides were later cross-checked in the laboratory. Approximately 15 μl of urine was used for each CCA urine lateral flow test, which was read on the spot after the prescribed incubation time and cross-checked by another observer. Results were recorded as 'negative', 'trace', 'single positive', 'double positive' and 'triple positive'.

### Data analysis

Prior to analysis, the raw dataset was cleaned to remove any children whose reported age was greater than 15. Prevalence estimates and 95% CI were calculated using version 2.8.0 of the R statistical package [21], along with all statistical modelling.

### Ethical approvals

The parents or guardians (head teachers) of all participating children were asked to sign written consent forms prior to their child's inclusion in the survey; in addition, each child was individually asked for oral consent. Each child was treated with praziquantel (40 mg/kg) and albendazole (400 mg) by the community nurse after taking part in the study.

Ethical clearance for this study was granted by the National Health System Local Research Ethics Committee at St Mary's Hospital in London and the Uganda Ministry of Health in Kampala.

## Abbreviations

CCA: circulating cathodic antigen; CI: confidence intervals; EPG: eggs per gram; NPV: negative predictive value; PPV: positive predictive value; RDTs: rapid diagnostic tests; SEA-ELISA: soluble egg antigen enzyme-linked immunosorbent assay; SP: specificity; SS: sensitivity; WHO: World Health Organization.

## Competing interests

The authors declare that they have no competing interests.

## Authors' contributions

This study was conceived by CJS, NBK and JRS, and the data collected during fieldwork in which all authors participated, with FK, NBK and JRS leading different teams. The data was compiled by MA and NBK, analysed by CJS and JRS and written up by CJS and JRS. All authors approved the final manuscript.
